# Exploration and visualization of gene expression with neuroanatomy in the adult mouse brain

**DOI:** 10.1186/1471-2105-9-153

**Published:** 2008-03-18

**Authors:** Christopher Lau, Lydia Ng, Carol Thompson, Sayan Pathak, Leonard Kuan, Allan Jones, Mike Hawrylycz

**Affiliations:** 1 Allen Institute for Brain Science, Seattle, WA, USA

## Abstract

**Background:**

Spatially mapped large scale gene expression databases enable quantitative comparison of data measurements across genes, anatomy, and phenotype. In most ongoing efforts to study gene expression in the mammalian brain, significant resources are applied to the mapping and visualization of data. This paper describes the implementation and utility of Brain Explorer, a 3D visualization tool for studying *in situ *hybridization-based (ISH) expression patterns in the Allen Brain Atlas, a genome-wide survey of 21,000 expression patterns in the C57BL\6J adult mouse brain.

**Results:**

Brain Explorer enables users to visualize gene expression data from the C57Bl/6J mouse brain in 3D at a resolution of 100 μm^3^, allowing co-display of several experiments as well as 179 reference neuro-anatomical structures. Brain Explorer also allows viewing of the original ISH images referenced from any point in a 3D data set. Anatomic and spatial homology searches can be performed from the application to find data sets with expression in specific structures and with similar expression patterns. This latter feature allows for anatomy independent queries and genome wide expression correlation studies.

**Conclusion:**

These tools offer convenient access to detailed expression information in the adult mouse brain and the ability to perform data mining and visualization of gene expression and neuroanatomy in an integrated manner.

## Background

Several efforts are underway to spatially identify gene expression in the mammalian central nervous system on a genomic scale [1-5]. Of the many techniques including *in situ *hybridization (ISH), microarray, SAGE [6] and its variants, and Q-PCR, colormetric non-radioactive ISH offers amongst the best alternatives for visualization of spatial localization of signal in its original setting. Whereas radioactive ISH has been cited [3] to have higher sensitivity for genes expressed at lower levels and a stronger signal-to-noise ratio than non-radioactive probes, the benefits of colormetric ISH for anatomic and tissue recognition as well as morphological cell characteristics is strong. These latter qualities are essential in the development of image registration, mapping, and visualization techniques enabling quantitative cross gene comparison.

Mapping expression patterns into a common frame of reference allows searches based on expression in identified anatomic regions as well as various statistical analyses [7-9]. Neuro-anatomical brain atlases provide a 2D means of identifying expression patterns, but analysis has been for the most part manual, qualitative, and labor intensive. By registering neuro-anatomical structures and data of interest into the same reference space, it is also possible to visualize and compare expression patterns in 3D. However, recent efforts to characterize genome-wide expression patterns across the entire brain at cellular resolution have resulted in data sets too large to be manually reconstructed and quantified [3]. Also, limiting the usage of 3D visualization has been the lower resolution of 3D data relative to 2D histological sections due to limits in acquisition and image processing.

We present a software application to visualize the Allen Reference Atlas [10] and Allen Brain Atlas (ABA) [11,12] simultaneously in three-dimensional space. Several examples are described to demonstrate its utility for both examining small sets of genes in detail as well as navigating the entire atlas of up to 21,000 genes. Our application also links the 3D representation of gene expression with the original full resolution 2D tissue sections. An area of interest in the 3D model can instantly link to the full resolution image for corroboration with the 3D model as well as detailed examination of subtle expression patterns. The Brain Explorer application is linked directly to the search facilities of the ABA database, which includes a voxel-based spatial homology search. Genes with high spatial expression correlation to a chosen seed gene expression pattern can be identified subject to a given user-identified region of interest. Gene expression identified by these means can be immediately viewed in Brain Explorer. The result is a powerful public domain desktop search and visualization application that is directly linked with the Allen Brain Atlas.

## Implementation

### Tissue Preparation

Gene localization data across the mouse brain were obtained for 21,000 genes using a semi-automated *in situ *hybridization (ISH) process as described in [12]. In brief, 25 μm thick tissues sections, at 100 or 200 μm intervals, were generated from 8-week old male C57BL/6J mice. Digoxigenin (DIG)-labeled riboprobes were synthesized and hybridized to mRNA transcripts in each tissue section. Tissue sections were scanned using Leica DM6000B microscopes and Leica DC500 cameras at 10× magnification, resulting in a resolution of 0.95 μm/pixel.

### Reference Atlas

All ISH tissue section images were registered against a reference atlas, which was generated using 528 25 μm thick Nissl-stained sections in the coronal plane [10]. Out of the 528 Nissl-stained sections, 132 sections at 100 μm spacing were annotated on the left half of the brain. All 528 reference atlas tissue sections were then assembled into a 3D volume using a rigid transformation at a resolution of 25 μm/pixel [9,13]. Using the same transformations, the annotations were also assembled into a 3D annotation volume consisting of 179 neuro-anatomical structures. In this volume, the non-annotated sections were filled in by shape interpolation. The goal was to obtain a 3D lofted anatomically sound version of the 2D Allen Reference Atlas plates.

The 3D annotation volume was transformed into a set of meshes representing the surfaces of the anatomic structures for display. Since the annotations were done on a section by section basis in 2D, several smoothing steps were necessary to make the 3D presentation more uniform in appearance. First, the volume was smoothed on a structure by structure basis using a level set curvature flow method [14] and small holes closed using morphological operations [15]. These operations were performed using the Insight Toolkit [16-18]. The structure surfaces were then extracted using marching cubes [19] and the resulting meshes low pass filtered for both smoothness and a reduction in resources required for storage and display. Finally, the meshes were decomposed into triangle strips for optimal display speed. This portion of the processing was performed using the Visualization Toolkit [20].

### Reference Atlas to ISH Mapping and ISH Quantification

The 3D reference atlas space was partitioned into regular 100 μm^3 ^grid cells (or voxels). For each gene expression experiment, the reference atlas was warped onto each ISH tissue section to preserve the anatomic fidelity of the ISH images. The registration used a rigid alignment followed by deformable refinements [9] and was performed with no user interaction across the entire ABA dataset. The 100 μm^3 ^grid was also warped into ISH image space using the deformations found by the registration. A fully automatic algorithm was developed to detect the expression signal in each tissue section [9]. Local image statistics were calculated over each grid cell (detected object count, average intensity, average object size, and fraction of the total grid cell area occupied by signal). The measurements and grid cell locations were recorded in a file for each gene experiment. These files are publicly available from the ABA website [11]. As in [9], we will refer to each 100 μm^3 ^grid cell and its related measurements as a *quadrat*.

### Reference Atlas-Based Search

The statistics for each quadrat were pooled over their corresponding neuro-anatomical regions and these measurements exposed as searchable values from the ABA website [9]. Expression level, density, and pattern (regional or uniform) can be queried in a dozen anatomic regions. Conjunctive or disjunctive (AND/OR) searches can be performed on up to three simultaneous structures. The search returns are ranked by specificity of expression, which is determined by the ratio of expression density in the structure of interest to the expression density in an enclosing structure. The search engine can be accessed via a web service [21].

### Spatial Homology Search

Performing quadrat-level processing independently of specific neuroanatomy forms the basis for searching by expression pattern in an image. Given an initial seed gene experiment (i.e. a specific expression pattern of interest on a given series of ISH images), it is possible to find other experiments with similar expression patterns. First, due to the tissue sectioning frequency, the 100 μm^3 ^grid was subsampled to 200 μm^3 ^in order to obtain sufficient overlap between experiments for comparison. For each 200 μm^3 ^quadrat, a measure we define as expression energy E(C) was calculated.

$$E(C)=\frac{\sum_{\forall p\in C}M(p)\times I(p)}{\left|C\right|},$$

where p is an image pixel that intersects quadrat C, |C| is the total number of pixels that intersect C, M(p) is the expression segmentation mask, which is either 1 for a pixel classified as gene expression or 0 for all other pixels, and I(p) is the grayscale value of the ISH image intensity (Intensity = 0.3·Red + 0.59·Green + 0.11·Blue). This measure can be robustly computed over all regions of the brain, and it essentially combines the features of expression intensity and expression density into a single measurement.

Pairs of gene experiments were then compared using Pearson's correlation coefficient to compute a similarity score:

$$CC(X,Y)=\frac{N\sum XY-\sum X\sum Y}{\sqrt{\left[N\sum X^{2}-{\left(\sum X\right)}^{2}\right]\left[N\sum Y^{2}-{\left(\sum Y\right)}^{2}\right]}}$$

where X(C) and Y(C) are respectively the expression energy at quadrat C for image series g_X _and g_Y_. Summation is over all quadrats within a spatial domain (for example, over one hemisphere or only within the neocortex) and N is the number of quadrats spanning the domain. The correlation coefficient quantifies the quality of a linear least squares fitting between the energy signal of the two gene experiments.

For each gene image series, the correlation coefficient with all other image series was calculated. All the image series were then sorted in descending order by the correlation coefficient, and the top 500 correlates were then stored in a database. Queries into this database are exposed via a web service [21].

### Desktop Application Implementation

A desktop application called *Brain Explorer *was developed to display the reference atlas and quadrat data in 3D. Brain Explorer is freely available for Microsoft Windows and Mac OS X from the ABA website [11]. Operating in a standalone mode, Brain Explorer can be used to view the Allen Reference Atlas, ISH gene experiment quadrat data files, or both simultaneously. With an active Internet connection, Brain Explorer can also be used to search for and download quadrat data files, perform spatial homology searches, and view the ISH data associated with each quadrat. Quadrat data files can also be downloaded from the ABA using a web browser.

The Brain Explorer code consists of cross-platform components for data handling and graphics written in C++ and using the OpenGL 1.1 API. Platform-specific user interface and networking components were written using Microsoft Foundation Classes for Windows (Microsoft Corporation, Redmond) and Cocoa for Mac OS X (Apple, Inc, Cupertino). ISH images and quadrat data are downloaded from the ABA [11] via HTTP. The built-in search functionality uses a SOAP web service interface at [21] to communicate with the ABA web application.

### Features and User Interface

**Figure 1:**
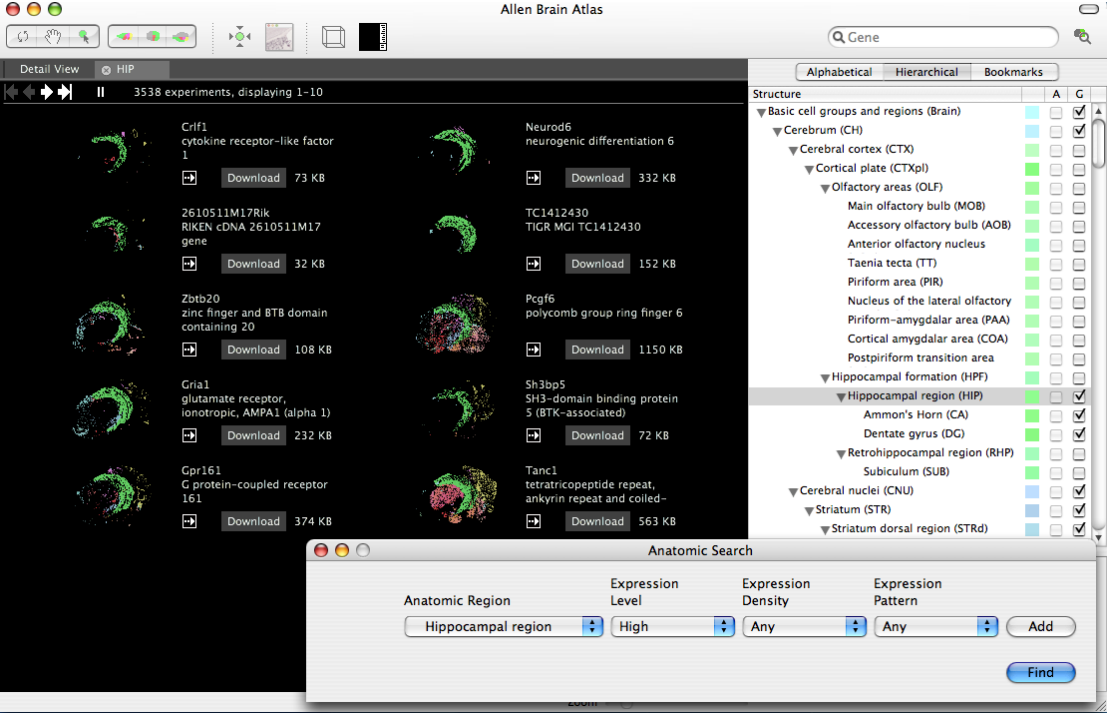
**List view mode**. 3D thumbnail images are shown representing the results from a search for genes with high density expression in the hippocampal region (shown in green) relative to other structures. Expression in the cerebral cortex was hidden so that the hippocampus can be seen. Each of the thumbnail data sets dynamically rotates about its y axis continuously to show the data from various angles. The anatomic search interface is shown in the bottom right hand corner.

Brain Explorer has two main display modes, list and detail mode. The list mode shows 3D thumbnail images (which rotate in the actual application) for several experiments side by side. The list can be constructed from performing a search on the Allen Brain Atlas by gene name, gene symbol, anatomic region, or similarity to a given seed experiment. Alternatively, the list can be loaded from a plain text file. For example, curated lists of genes with enriched expression in various brain regions can be exported from the ABA website [11] and loaded into Brain Explorer. In figure 1, a search was performed for genes with high density expression in the hippocampal region relative to other structures. Quadrats containing 5 or more expressing objects are displayed as a single pixel in the thumbnail image, and the pixels are colored according to the quadrats' anatomic annotations. The colors are defined by the reference atlas as shown on the right hand side of figure 1. Users can choose multiple experiments from this view to examine in the "Detail View" mode.

**Figure 2:**
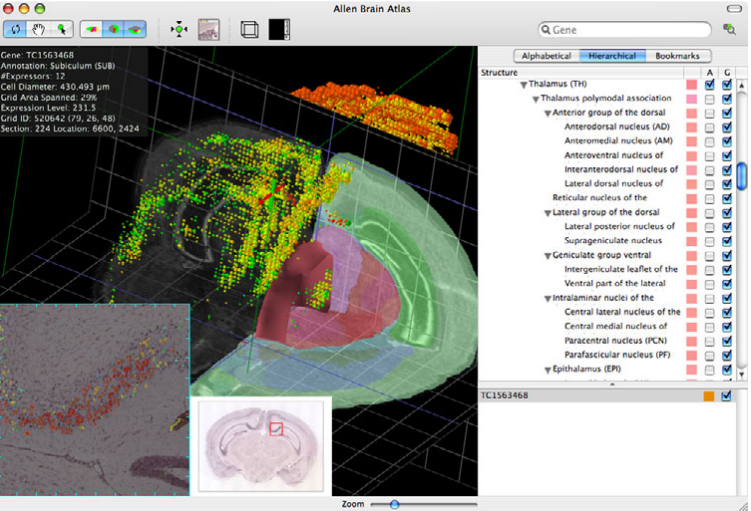
**Main Brain Explorer display mode**. Expression of TC1563468 (*Etv1*, transcription factor variant gene 1) is shown with the quadrats colored by expression level (blue-green is low, yellow is medium, and red is high). The images at the bottom left corner show the original image data from which the data for a quadrat in the subiculum were measured along with some of the surrounding tissue. The detected signal, color mapped by expression level, is shown blended with the original image. The tick marks on the ISH image indicate 100 μm intervals, and the markings on the reference atlas Nissl sections indicate 1 cm.

The detail display mode of Brain Explorer can show one or more experiments in the same space along with the reference atlas (figure 2). In this mode, spheres are used to represent quadrats, with the size of the spheres directly proportional to the number of objects detected in each quadrat. The color of the spheres can be uniform for each gene (ideal for comparing two or more genes), colored according to anatomic annotation, or colored according to expression level. The reference atlas Nissl and annotation volumes can be overlaid and cross sections in the coronal, sagittal, and horizontal planes displayed. The 3D reference atlas meshes can be displayed in any combination as well. The 3D objects in this mode are fully interactive.

The Brain Explorer application enables the following mouse click based operations:

• Clicking and dragging the reference atlas Nissl sections repositions the section faces.

• Clicking on a brain structure (either the 3D mesh or an annotated Nissl section) displays the name of the structure and its location in the atlas ontological hierarchy.

• Clicking on a quadrat shows the portion of the original ISH image from which the quadrat measurements were made.

• Double-clicking on the ISH section opens a window in which the actual high resolution section from the Allen Brain Atlas can be examined.

The 3D view can also be manipulated using standard 3D controls such as rotating, panning, zooming, and applying orthogonal clipping planes (i.e., defining a cube of interest and excluding all objects outside of the cube from view). In addition to using clipping planes, the visible quadrats can be filtered according to quadrat values (number of objects and expression level) and anatomic location. Any of the view settings described above can be saved as bookmarks for restoration at a later time. Users can optionally animate the view when restoring bookmarks for a smooth transition to the bookmarked view state.

## Results

The ability to perform search and visualization on a commonly mapped dataset of genomic scale should greatly increase the power and benefit of neuroinformatics to researchers. The following specific scenarios illustrate this concept.

**Figure 3:**
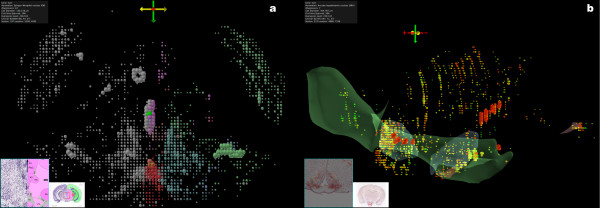
**Expression of *Cart***. Coronal and off-axis sagittal views of *Cart*, showing distinct patterns of expression in the somatosensory cortex, accumbens, arcuate nucleus, nucleus of the solitary tract, Edinger-Westphal nucleus, piriform area, and main olfactory bulb. In (a), the quadrat colors correspond to the reference atlas colors of the structures in which they were mapped. A quadrat in the Edinger-Westphal nucleus was selected and the corresponding reference atlas plate displayed. Expression in the right half of the brain was hidden in (b) and the quadrat colors are mapped to the average expression level. Also in (b), several colored transparent meshes for neuro-anatomical structures are shown according to the reference atlas color coding. The olfactory areas are green, nucleus accumbens and striatum-like amgydalar nuclei are blue, the nucleus of the solitary tract is purple, and the arcuate hypothalamic nucleus is red.

Cocaine and amphetamine-regulated transcript (*Cart*) has been identified as a neuropeptide involved in reward and reinforcement circuitry [22,23]. Figure 3 shows *Cart *expression in areas related to this circuitry, the nucleus accumbens and amygdalar nuclei. *Cart *also expresses in the somatosensory cortex, olfactory areas, and nuclei of the solitary tract, suggesting a role in sensory processes and autonomic control [23]. Expression in the arcuate nucleus is supported by *Cart*'s role as a satiety factor [24].

**Figure 4:**
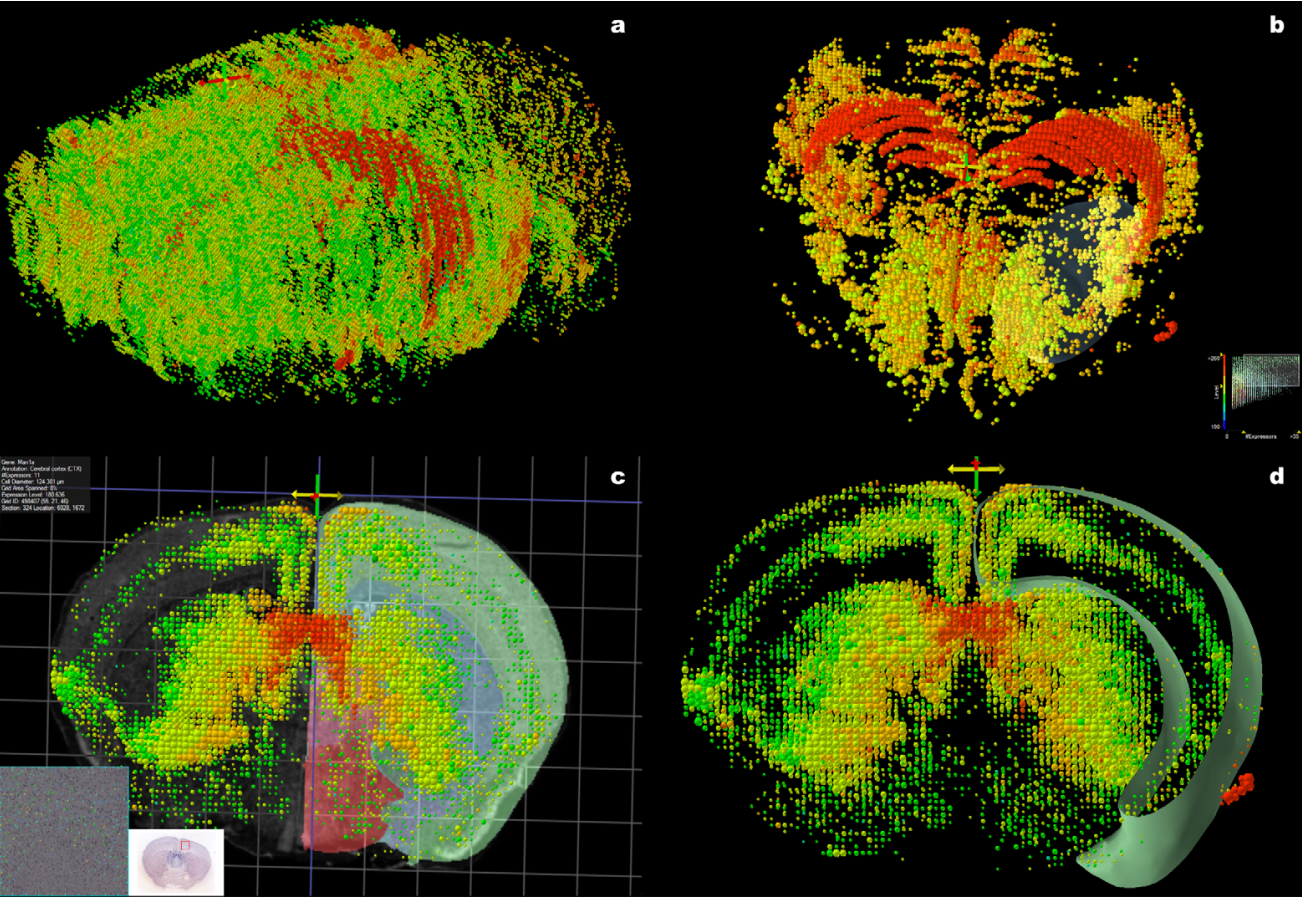
**Expression of *Man1a***. *Man1a *expresses densely in the cortex, making it difficult to visualize expression deeper inside the brain. The view with no filtering is shown in (a). Low density and low level expression is hidden in (b) to emphasize the striatum and hippocampus – the lower right hand corner shows the thresholding control. In (c), a coronal clipping plane is used to hide expression from roughly the striatum forward. A coronal atlas section is shown behind the striatum to hide expression towards the back of the brain. The combination of clipping plane and atlas section reveals expression restricted to a layer of the cortex. A similar result can be achieved by setting both front and back clipping planes and showing the outer surface of the cortex (d).

Alpha-mannosidosis is a lysosomal storage disease with autosomal recessive inheritance associated with disruption to the gene mannosidase 1, alpha (*Man1a*). It has severe neuropsychological and psychopathological complications [25,26]. The gene *Man1a *expresses in many areas of the cortex, making it potentially difficult to view interior structures, such as the hippocampus where expression is comparatively strong. Since the hippocampus is expressing very highly and the cortex relatively lowly in *Man1a*, one option is to hide the quadrats with lower levels of expression (figure 4b). Another option is to simulate cross sections using the cutting plane option. In figure 4c and 4d, coronal cross sections reveal layering in the cortex.

**Figure 5:**
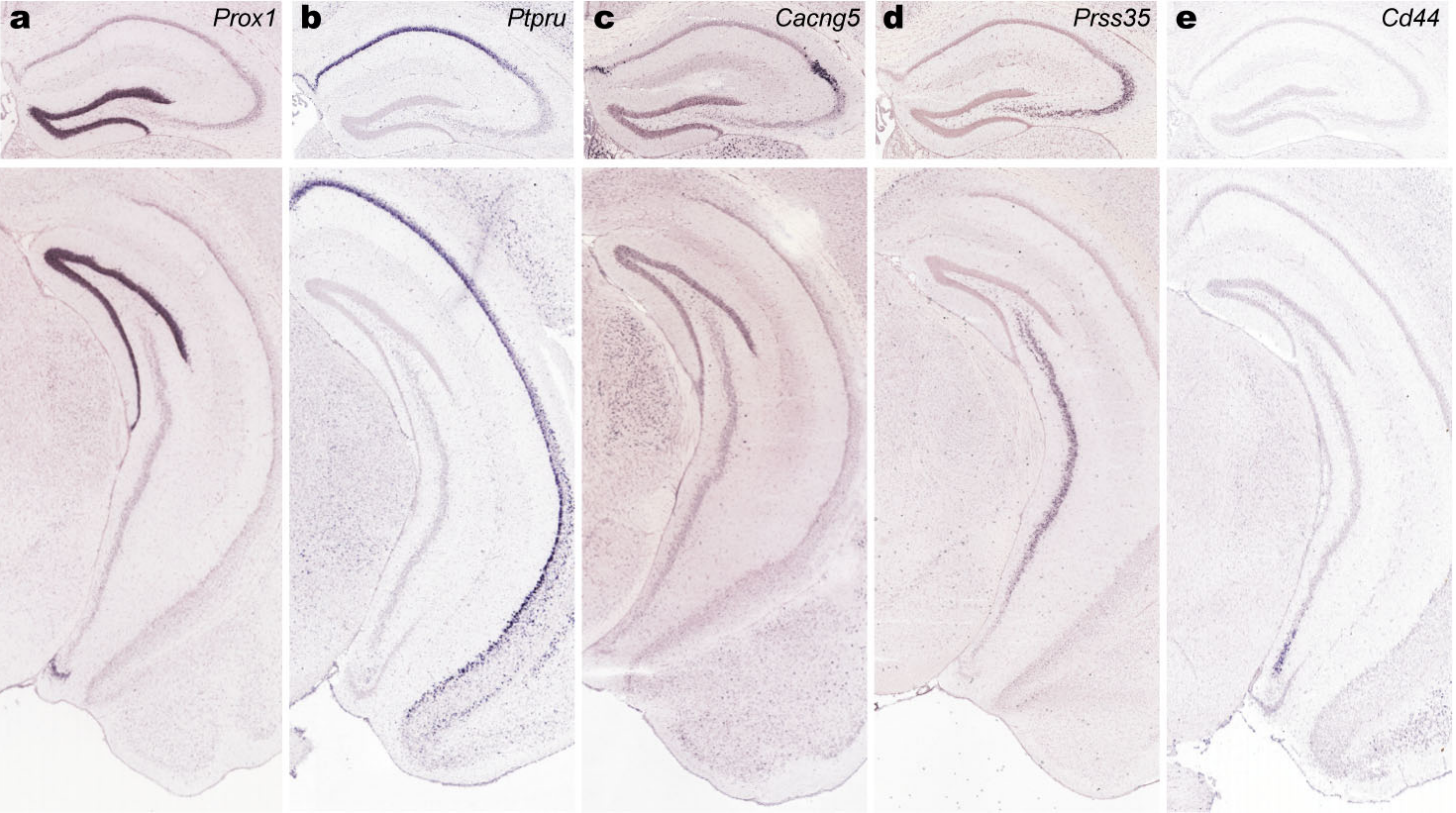
**Spatial homology search seed image series**. Representative ISH sections are shown for hippocampal field-specific markers. These image series were used as seeds for the spatial homology searches shown in Figure 6: Prox1 for the dentate gyrus (a), *Ptpru *for CA1 (b), *Cacng5 *for CA2 (c), *Prss35 *for CA3 (d) and *Cd44 *for the ventral CA3 (e).

The hippocampus is known to play a critical role in learning and memory [27], and its subregions have been well characterized by morphology, electrophysiology and connectivity. The hippocampus is generally divided into two regions: the granule cells of the dentate gyrus (DG), and the excitatory pyramidal neurons of Ammon's Horn, which is further subdivided into CA1, CA2, and CA3. Transcriptional profiling has shown these regions to be molecularly distinct [28,29], and this differential gene expression is presumably the basis for known field-specific functional differentiation. The ABA ISH dataset has provided novel examples of field-specific molecular markers, such as *Prox1 *for the DG, *Ptpru *for CA1, *Cacng5 *for CA2, and *Prss35 *for CA3 (figure 5a–d). Furthermore, genomic scale gene expression profiling has the potential of revealing finer, presumably functional, subdivisions which cannot be distinguished solely by cytoarchitecture [12]. *Cd44 *is an example of a gene delineating the boundaries of a novel ventral domain in CA3 (figure 5e), which appears to be complementary to *Prss35 *in (d).

**Figure 6:**
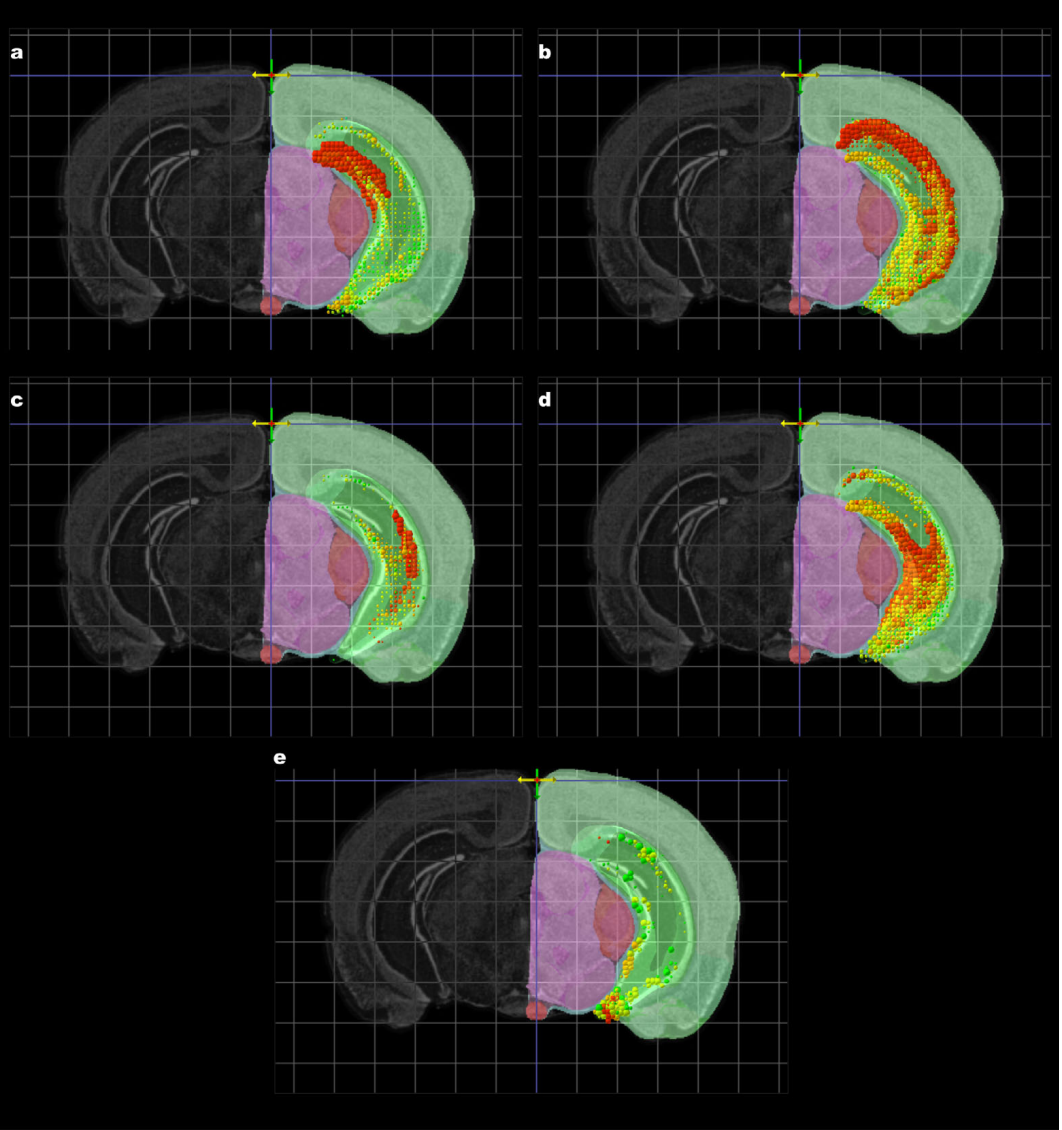
**Top 10 results for a set of spatial homology searches are shown overlaid for subregions of the hippocampus**. The seeds used are shown in Figure 5: Prox1 for the dentate gyrus (a), *Ptpru *for CA1 (b), *Cacng5 *for CA2 (c), *Prss35 *for CA3 (d) and *Cd44 *for the ventral CA3 (e). The view is restricted to the hippocampal region. A rostral clipping plane and coronal atlas plate were set to isolate the middle of the hippocampal region. The 3D projections can be compared with the hippocampal structures shown in the Nissl-stained reference atlas on the left hand side of the figures.

**Figure 7:**
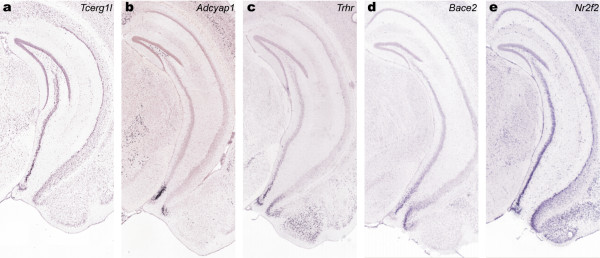
**Spatial homology search with gene *Cd44***. Top 5 returns from a spatial homology search with gene *Cd44 *(Figures 5e and 6e) demonstrating co-expression of the ventral CA3 tip with the ventral tip of DG and CA1.

Three-dimensional spatial homology searches and 3D visualization of these genomic ISH datasets can assist with identifying and categorizing gene expression patterns. These hippocampal field-specific markers for major subfields DG, CA1, CA2, and CA3, as well as the subfield marker for the ventral tip of CA3 (figure 5a–e) were used as seed genes for a spatial homology search within the hippocampus (figure 6a–e). Gene expression statistics for the seed and the top 10 results superimposed in expression level color mode are displayed in figure 6. The high concentration of red and orange quadrats shows that the top results have similar differential enrichment patterns to the seed. 3D visualization using expression level color also reveals a dorsal-ventral gradient in the CA3-enriched genes in panel (d): the dorsal areas are predominately red-orange and the ventral areas yellow-green. Panel (e) shows the search result with a seed gene enriched in the ventral tip of CA3 (*Cd44*). The top results also exhibit differential enrichment in the ventral region and closer inspection of the ISH images (figure 7) reveals co-expression of the ventral CA3 tip with the ventral tip of the dentate gyrus and CA1. These dorsoventral domains indicated by *Prss35, Cd44*, and their spatial homologues, are consistent with observed functional differentiation in the hippocampus. Specifically, the dorsal half or two-thirds of the hippocampus is required for proper spatial learning and memory, while the ventral half or one-third of the hippocampus is associated with anxiety-related behaviors [30,31].

## Discussion

The most common way to visualize gene expression in 3D has been using general-purpose reconstruction and visualization software packages. A 3D volume is reconstructed from serial sections, the signal is segmented, and polygonal surfaces are extracted from the segmentation. Each of these steps is typically a manual process and can result in a high quality 3D model when done carefully. In order to aid interpretation and comparison of data sets, mapping gene expression data into a common spatial framework is desirable. Two projects have made progress into this area. The Edinburgh Mouse Atlas Project has developed interactive tools to segment and map expression data to a set of developmental atlases [32,33]. Users can submit data to the Edinburgh Mouse Atlas Gene Expression Database, which can be queried by anatomical ontology or an arbitrary region drawn on one of the atlases [8]. The Mouse Atlas Project similarly allows contributors to map gene expression data to a reference adult C57BL/6J brain atlas using a provided set of tools [34,35]. Both projects have developed visualization software to view cross sections of image volumes, 3D surfaces, and the organization of neuro-anatomical ontologies.

The Allen Brain Atlas represents a collection of gene expression patterns for approximately 27,000 experiments and 700,000 images. Three-dimensional reconstructions are readily available for all of the data. Using Brain Explorer to view the 3D data, users can quickly see the complete distribution of expression across the brain in each data set at once without having to examine each image section. Three-dimensional expression patterns spanning multiple brain sections can also be more easily appreciated. This is particularly useful in complex structures such as the hippocampus. The reconstructed data is all in the reference atlas coordinate space, allowing multiple data sets to be viewed simultaneously for direct comparison. Spatial homology searches can be performed from anywhere in the Brain Explorer application to quickly find experiments with similar expression patterns. With appropriate seed genes, this type of search can be used to examine subdivisions of neuroanatomic structures delineated by gene expression.

Data similar in nature to the ABA gene expression data are typically visualized by either building polygonal meshes from the data or directly rendering 3D reconstructed volumes. Rendering quadrats as spheres enables several features to be implemented easily on common inexpensive graphics hardware. One of the most important features is easy access to the original ISH data for a quadrat. The sphere approach allows quadrats to be targeted quickly and accurately with the mouse cursor, which displays the original section as well as a summary of the expression measured at that quadrat. Double-clicking on the section image opens up the section in a separate window and allows unlimited navigation within the section as well as across the series of sections. This direct level of access to the original data also reduces the impact of inaccuracies in the automated registration and segmentation methods. Anatomical relationships inferred from the global 3D view can be quickly confirmed and refined on the original ISH images.

Rendering quadrats as spheres also allows multiple variables to be displayed by varying the size and color of the spheres. The current implementation maps density of expression to size and optionally the user's choice of anatomic annotation or expression level to the color. However, mapping other parameters is also possible. Another use of the quadrat values is filtering of the quadrats displayed, and the sphere visualization method allows the filtering to be adjusted interactively. Quadrats can be filtered according to neuro-anatomical structure boundaries as well as by quadrat values. The examples we have presented show various scenarios in which filtering was used to reveal different areas of interest in the display.

The use of spatial homology searches to identify large sets of genes with expression profiles similar to a dorsal or ventral hippocampal seed gene out of the genomic ABA dataset functions like a transcriptional profiling experiment of individual hippocampal domains. The results of such searches could yield insight into the molecular differences underlying functional differentiation across the hippocampus. Additionally, the seed genes and resulting spatial homologue lists provide potential insight into transcriptional regulation. *Prox1*, the DG seed gene in figure 6, is a transcriptional factor, and the spatial homologues derived from this search could present potential downstream targets for this transcription factor. Conversely, the seed gene *Cd44 *yielded a list of ventrally enriched genes in CA3 which included the transcription factor *Nr2f2*. The precise coexpression of a transcription factor with other similarly expressed genes could provide a way of screening transcription factors and their downstream targets for future analyses of transcriptional regulation.

## Conclusion

We have developed a desktop application, Brain Explorer, to view data from the Allen Reference and Brain Atlases in 3D. The Brain Explorer application is closely linked with the web-based ABA database, which contains 3D reconstructions for over 27,000 data sets spanning the entire mouse genome. In addition to being linked with the 3D search facility of  the Allen Brain Atlas, Brain Explorer incorporates a 3D expression  homology search for identifying highly correlated genes with the user's  input. Additionally, for any part of the  3D model, immediate access to the original ISH images is provided. Together these tools offer unprecedented access to detailed expression information in the adult mouse brain and the ability to perform data mining and visualization of gene expression and neuroanatomy in an integrated manner.

## Availability and requirements

• **Project name**: Brain Explorer

• **Project home page**: 

• **Operating system(s)**: Windows XP and above, Mac OS X 10.3.9 and above

• **Programming languages**: C++, Objective-C

• **Other requirements**: OpenGL hardware-accelerated graphics card

• **License**: Allen Brain Atlas 

• **Any restrictions to use by non-academics**: None

## Authors' contributions

CL designed and implemented the Brain Explorer application. LN developed the registration and spatial homology search algorithms. SP and MH were the primary designers of the anatomic search algorithm. LK and SP developed the ISH image segmentation and quantification algorithms. CT analyzed the spatial homology search results for the hippocampus. CL, LN, CT, and MH wrote the manuscript. AJ provided overall project vision and support. All authors read and approved the final manuscript.

## References

[B1] Carninci P, Kasukawa T, Katayama S, Gough J, Frith MC, Maeda N, Oyama R, Ravasi T, Lenhard B, Wells C, Kodzius R, Shimokawa K, Bajic VB, Brenner SE, Batalov S, Forrest ARR, Zavolan M, Davis MJ, Wilming LG, Aidinis V, Allen JE, Ambesi-Impiombato X, Apweiler R, Aturaliya RN, Bailey TL, Bansal M, Baxter L, Beisel KW, Bersano T, Bono H, Chalk AM, Chiu KP, Choudhary V, Christoffels A, Clutterbuck DR, Crowe ML, Dalla E, Dalrymple BP, de Bono B, Della Gatta G, di Bernardo D, Down T, Engstrom P, Fagiolini M, Faulkner G, Fletcher CF, Fukushima T, Furuno M, Futaki S, Gariboldi M, Georgii-Hemming P, Gingeras TR, Gojobori T, Green RE, Gustincich S, Harbers M, Hayashi Y, Hensch TK, Hirokawa N, Hill D, Huminiecki L, Iacono M, Ikeo K, Iwama A, Ishikawa T, Jakt M, Kanapin A, Katoh M, Kawasawa Y, Kelso J, Kitamura H, Kitano H, Kollias G, Krishnan SPT, Kruger A, Kummerfeld SK, Kurochkin IV, Lareau LF, Lazarevic D, Lipovich L, Liu J, Liuni S, McWilliam S, Babu MM, Madera M, Marchionni L, Matsuda H, Matsuzawa S, Miki H, Mignone F, Miyake S, Morris K, Mottagui-Tabar S, Mulder N, Nakano N, Nakauchi H, Ng P, Nilsson R, Nishiguchi S, Nishikawa S, Nori F, Ohara O, Okazaki Y, Orlando V, Pang KC, Pavan WJ, Pavesi G, Pesole G, Petrovsky N, Piazza S, Reed J, Reid JF, Ring BZ, Ringwald M, Rost B, Ruan Y, Salzberg SL, Sandelin A, Schneider C, Schonbach C, Sekiguchi K, Semple CAM, Seno S, Sessa L, Sheng Y, Shibata Y, Shimada H, Shimada K, Silva D, Sinclair B, Sperling S, Stupka E, Sugiura K, Sultana R, Takenaka Y, Taki K, Tammoja K, Tan SL, Tang S, Taylor MS, Tegner J, Teichmann SA, Ueda HR, van Nimwegen E, Verardo R, Wei CL, Yagi K, Yamanishi H, Zabarovsky E, Zhu S, Zimmer A, Hide W, Bult C, Grimmond SM, Teasdale RD, Liu ET, Brusic V, Quackenbush J, Wahlestedt C, Mattick JS, Hume DA, Kai C, Sasaki D, Tomaru Y, Fukuda S, Kanamori-Katayama M, Suzuki M, Aoki J, Arakawa T, Iida J, Imamura K, Itoh M, Kato T, Kawaji H, Kawagashira N, Kawashima T, Kojima M, Kondo S, Konno H, Nakano K, Ninomiya N, Nishio T, Okada M, Plessy C, Shibata K, Shiraki T, Suzuki S, Tagami M, Waki K, Watahiki A, Okamura-Oho Y, Suzuki H, Kawai J, Hayashizaki Y (2005). The transcriptional landscape of the mammalian genome. Science.

[B2] Gong S, Zheng C, Doughty ML, Losos K, Didkovsky N, Schambra UB, Nowak NJ, Joyner A, Leblanc G, Hatten ME, Heintz N (2003). A gene expression atlas of the central nervous system based on bacterial artificial chromosomes. Nature.

[B3] Magdaleno S, Jensen P, Brumwell CL, Seal A, Lehman K, Asbury A, Cheung T, Cornelius T, Batten DM, Eden C, Norland SM, Rice DS, Dosooye N, Shakya S, Mehta P, Curran T (2006). BGEM: An in situ hybridization database of gene expression in the embryonic and adult mouse nervous system. PLoS Biology.

[B4] Sandberg R, Yasuda R, Pankratz DG, Carter TA, Del Rio JA, Wodicka L, Mayford M, Lockhart DJ, Barlow C (2000). Regional and strain-specific gene expression mapping in the adult mouse brain. Proceedings Of The National Academy Of Sciences Of The United States Of America.

[B5] Su AI, Cooke MP, Ching KA, Hakak Y, Walker JR, Wiltshire T, Orth AP, Vega RG, Sapinoso LM, Moqrich A, Patapoutian A, Hampton GM, Schultz PG, Hogenesch JB (2002). Large-scale analysis of the human and mouse transcriptomes. Proceedings Of The National Academy Of Sciences Of The United States Of America.

[B6] Velculescu VE, Madden SL, Zhang L, Lash AE, Yu J, Rago C, Lal A, Wang CJ, Beaudry GA, Ciriello KM, Cook BP, Dufault MR, Ferguson AT, Gao YH, He TC, Hermeking H, Hiraldo SK, Hwang PM, Lopez MA, Luderer HF, Mathews B, Petroziello JM, Polyak K, Zawel L, Zhang W, Zhang XM, Zhou W, Haluska FG, Jen J, Sukumar S, Landes GM, Riggins GJ, Vogelstein B, Kinzler KW (1999). Analysis of human transcriptomes. Nature Genetics.

[B7] Carson JP, Ju T, Lu HC, Thaller C, Xu M, Pallas SL, Crair MC, Warren J, Chiu W, Eichele G (2005). A Digital Atlas to Characterize the Mouse Brain Transcriptome. PLoS Computational Biology.

[B8] Christiansen JH, Yang Y, Venkataraman S, Richardson L, Stevenson P, Burton N, Baldock RA, Davidson DR (2006). EMAGE: a spatial database of gene expression patterns during mouse embryo development. Nucleic Acids Research.

[B9] Ng L, Pathak S, Kuan L, Lau C, Dong H, Sodt A, Dang C, Avants B, Yushkevich P, Gee J, Haynor D, Lein ES, Jones A, Hawrylycz M (2007). Neuroinformatics for genome-wide 3-D gene expression mapping in the mouse brain. IEEE/ACM Transactions on Computational Biology and Bioinformatics.

[B10] Dong H (2008). The Allen Reference Atlas.

[B11] The Allen Brain Atlas. http://www.brain-map.org.

[B12] Lein ES, Hawrylycz MJ, Ao N, Ayres M, Bensinger A, Bernard A, Boe AF, Boguski MS, Brockway KS, Byrnes EJ, Chen L, Chen L, Chen TM, Chin MC, Chong J, Crook BE, Czaplinska A, Dang CN, Datta S, Dee NR, Desaki AL, Desta T, Diep E, Dolbeare TA, Donelan MJ, Dong HW, Dougherty JG, Duncan BJ, Ebbert AJ, Eichele G, Estin LK, Faber C, Facer BA, Fields R, Fischer SR, Fliss TP, Frensley C, Gates SN, Glattfelder KJ, Halverson KR, Hart MR, Hohmann JG, Howell MP, Jeung DP, Johnson RA, Karr PT, Kawal R, Kidney JM, Knapik RH, Kuan CL, Lake JH, Laramee AR, Larsen KD, Lau C, Lemon TA, Liang AJ, Liu Y, Luong LT, Michaels J, Morgan JJ, Morgan RJ, Mortrud MT, Mosqueda NF, Ng LL, Ng R, Orta GJ, Overly CC, Pak TH, Parry SE, Pathak SD, Pearson OC, Puchalski RB, Riley ZL, Rockett HR, Rowland SA, Royall JJ, Ruiz MJ, Sarno NR, Schaffnit K, Shapovalova NV, Sivisay T, Slaughterbeck CR, Smith SC, Smith KA, Smith BI, Sodt AJ, Stewart NN, Stumpf KR, Sunkin SM, Sutram M, Tam A, Teemer CD, Thaller C, Thompson CL, Varnam LR, Visel A, Whitlock RM, Wohnoutka PE, Wolkey CK, Wong VY, Wood M, Yaylaoglu MB, Young RC, Youngstrom BL, Yuan XF, Zhang B, Zwingman TA, Jones AR (2007). Genome-wide atlas of gene expression in the adult mouse brain. Nature.

[B13] Yushkevich PA, Avants BB, Zhang H, Burstein BD, Ng L, Hawrylycz M, Gee J (2005). Using MRI to build a 3D reference atlas of the mouse brain from histological images. Proc Intl Soc Magn Res Med.

[B14] Sethian JA (1997). Level Set Methods: Evolving Interfaces in Geometry, Fluid Mechanics, Computer Vision and Material Science.

[B15] Gonzalez R, Woods R (2002). Digital Image Processing.

[B16] Insight Toolkit. http://www.itk.org.

[B17] Ibanez L, Schroeder W, Ng L, Cates J (2005). The ITK Software Guide.

[B18] Yoo T (2004). Insight into Images.

[B19] Lorensen WE, Cline HE (1987). Marching cubes: A high resolution 3D surface construction algorithm.

[B20] Visualization Toolkit. http://www.vtk.org.

[B21] The Allen Brain Atlas Gene Search Web Service. http://www.brain-map.org/services/GeneSearchService?wsdl.

[B22] Elias CF, Lee CE, Kelly JF, Ahima RS, Kuhar M, Saper CB, Elmquist JK (2001). Characterization of CART neurons in the rat and human hypothalamus. Journal of Comparative Neurology.

[B23] Koylu EO, Couceyro PR, Lambert PD, Kuhar MJ (1998). Cocaine- and amphetamine-regulated transcript peptide immunohistochemical localization in the rat brain. Journal of Comparative Neurology.

[B24] Kristensen P, Judge ME, Thim L, Ribel U, Christjansen KN, Wulff BS, Clausen JT, Jensen PB, Madsen OD, Vrang N, Larsen PJ, Hastrup S (1998). Hypothalamic CART is a new anorectic peptide regulated by leptin. Nature.

[B25] D'Hooge R, R. LR, Beckers T, Balschun D, Schwake M, Reiss K, von Figura K, Saftig P (2005). Neurocognitive and psychotiform behavioral alterations and enhanced hippocampal long-term potentiation in transgenic mice displaying neuropathological features of human-mannosidosis. Journal of Neuroscience.

[B26] Stinchi S, Lullmann-Rauch R, Hartmann D, Coenen R, Beccari T, Orlacchio A, von Figura K, Saftig P (1999). Targeted disruption of the lysosomal alpha-mannosidase gene results in mice resembling a mild form of human alpha-mannosidosis. Human Molecular Genetics.

[B27] Anderson P, Morris R, Amaral D, Bliss T, O'Keefe J (2007). Historical perspective: Proposed functions, biological characteristics, and neurobiological models of the hippocampus. The Hippocampus Book.

[B28] Lein ES, Zhao XY, Gage FH (2004). Defining a molecular atlas of the hippocampus using DNA microarrays and high-throughput in situ hybridization. Journal Of Neuroscience.

[B29] Zhao X, Lein ES, He A, Smith SC, Aston C, Gage FH (2001). Transcriptional profiling reveals strict boundaries between hippocampus subregions. Transcriptional profiling reveals strict boundaries between hippocampus subregions.

[B30] Bannerman DM, Rawlins JNP, McHugh SB, Deacon RMJ, Yee BK, Bzst T, Zhang WN, Pothuizen HHJ, Feldon J (2004). Regional dissociations within the hippocampus-memory and anxiety. Neuroscience and Biobehavioral Reviews.

[B31] Moser MB, Moser EI (1998). Functional differentiation in the hippocampus. Hippocampus.

[B32] Sarma S, Kerwin J, Puelles L, Scott M, Strachan T, Feng G, Sharpe J, Davidson D, Baldock R, Lindsay S (2005). 3D modelling, gene expression mapping and post-mapping image analysis in the developing human brain. Brain Research Bulletin.

[B33] The Edinburgh Mouse Atlas Project. http://genex.hgu.mrc.ac.uk.

[B34] MacKenzie-Graham A, Lee E, Dinov ID, Bota M, Shattuck DW, Ruffins S, Yuan H, Konstantinidis F, Pitiot A, Ding Y, Hu G, Jacobs RE, Toga AW (2004). A multimodal, multidimensional atlas of the C57BL/6J mouse brain. Journal of Anatomy.

[B35] The Mouse Atlas Project. http://www.loni.ucla.edu/MAP.

